# Implementing Anti-Amyloid Therapies in Memory Clinics: A Qualitative Study of Clinician Experiences

**DOI:** 10.1093/geroni/igaf122.2524

**Published:** 2025-12-31

**Authors:** Joanna Paladino, Anna Parks, Daniel Dohan, Seth Gale, Liliana Ramirez Gomez, Christine Ritchie, Sachin Shah, Ayush Thacker

**Affiliations:** Massachusetts General Hospital, Boston, Massachusetts, United States; University of Utah, Salt Lake City, Utah, United States; University of California, San Francisco, San Francisco, California, United States; Brigham and Women’s Hospital, Boston, Massachusetts, United States; Massachusetts General Hospital, Boston, Massachusetts, United States; Massachusetts General Hospital, Boston, Massachusetts, United States; Massachusetts General Hospital, Boston, Massachusetts, United States; Massachusetts General Hospital, Boston, Massachusetts, United States

## Abstract

Anti-amyloid therapies (AATs) have changed the diagnosis and treatment paradigm of Alzheimer’s disease (AD). Our interdisciplinary team completed a qualitative study of clinicians’ experience with AAT implementation. We conducted semi-structured interviews with 27 prescribing clinicians at seven U.S. academic medical centers. We identified three themes using thematic content analysis. First, AATs affect practice norms for AD diagnosis. Clinicians feel added pressure for time-efficiency of the diagnostic process, sense expectations for more accurate diagnoses, and integrate AAT considerations into diagnostic disclosure and choice of tests. Even with increased time pressure, conversations about AATs are unfolding over multiple visits because of the time it takes for eligibility tests to return and to allow for shared decision-making. Second, the availability of AATs creates a paradigm shift with opportunities and challenges for clinicians. Clinicians describe feeling a sense of hope that is an ‘antidote’ to nihilism in dementia care. They also experience burdens from the increased workload and urgency of managing patients on infusion, inadequate institutional support, and reduced clinical time for patients not on AAT. Third, clinicians and institutions vary in lecanemab treatment protocols, including comfort with offering lecanemab to patients on concurrent anticoagulants or homozygous for ApoE4. While institutional protocols initially adhered closely to published appropriate use criteria regarding patient eligibility, clinicians reported modifications of eligibility criteria over time. Clinicians and clinical leaders can use findings from this study to consider structural changes to accommodate new workflows related to AAT and to inform the development of AAT decision-support interventions.

